# Structure-Based Evaluation of Hybrid Lipid–Polymer Nanoparticles: The Role of the Polymeric Guest

**DOI:** 10.3390/polym16020290

**Published:** 2024-01-20

**Authors:** Maria Chountoulesi, Natassa Pippa, Aleksander Forys, Barbara Trzebicka, Stergios Pispas

**Affiliations:** 1 Section of Pharmaceutical Technology, Department of Pharmacy, National and Kapodistrian University of Athens, Panepistimioupolis Zografou, 15771 Athens, Greece; mchountoules@pharm.uoa.gr; 2 Centre of Polymer and Carbon Materials, Polish Academy of Sciences, ul. M. Curie-Skłodowskiej 34, 41-819 Zabrze, Poland; aforys@cmpw-pan.pl (A.F.); btrzebicka@cmpw-pan.pl (B.T.); 3 Theoretical and Physical Chemistry Institute, National Hellenic Research Foundation, 48 Vassileos Constantinou Avenue, 11635 Athens, Greece

**Keywords:** phospholipids, block copolymers, nanoparticles, cryo-TEM, vesicles, micelles

## Abstract

The combination of phospholipids and block-copolymers yields advanced hybrid nanoparticles through the self-assembly process in an aqueous environment. The physicochemical features of the lipid/polymer components, like the lipid–polymer molar ratio, the macromolecular architecture of the block copolymer, the main transition temperature of the phospholipid, as well as the formulation and preparation protocol parameters, are some of the most crucial parameters for the formation of hybrid lipid/polymer vesicles and for the differentiation of their morphology. The morphology, along with other physicochemical nanoparticle characteristics are strictly correlated with the nanoparticle’s later biological behavior after being administered, affecting interactions with cells, biodistribution, uptake, toxicity, drug release, etc. In the present study, a structural evaluation of hybrid lipid–polymer nanoparticles based on cryo-TEM studies was undertaken. Different kinds of hybrid lipid–polymer nanoparticles were designed and developed using phospholipids and block copolymers with different preparation protocols. The structures obtained ranged from spherical vesicles to rod-shaped structures, worm-like micelles, and irregular morphologies. The obtained morphologies were correlated with the formulation and preparation parameters and especially the type of lipid, the polymeric guest, and their ratio.

## 1. Introduction

Drug delivery systems are widely used for the improvement of the pharmacological and pharmacokinetic profiles of active substances. Nanocarriers are drug delivery systems and are composed of different kinds of excipients, i.e., polymers, lipids, surfactants, inorganic materials, etc. Each class of nanocarriers utilized for drug delivery exhibits several advantages and limitations for clinical use. For this reason, the combination of different materials is an attractive methodology for the fast clinical translation of the drug delivery nanocarriers [1,2,3,4].

By combining phospholipids and block copolymers, hybrid nanoparticles can be formed in an aqueous environment. The molar ratio of the components, the molecular weight and macromolecular architecture of the block copolymer, the main transition temperature of the phospholipid, and the surface charge, accompanied by the selection of an appropriate preparation protocol, are the crucial parameters for the formation of hybrid lipid/polymer vesicles and other nanomorphologies [1,5,6,7,8,9]. Furthermore, spheroplexes are fabricated with hybrid PLGA-cationic lipid nanoparticles for in vitro and oral delivery of siRNA [10]. Hybrid nanoparticles composed of the biocompatible polymer poly(methyl methacrylate) (PMMA) and the cationic lipid dioctadecyl dimethyl ammonium bromide (DODAB) and produced via the emulsion polymerization of methyl methacrylate (MMA) monomer in the presence of DODAB were used as immunoadjuvants. They successfully encapsulated ovalbumin and induced both cellular and cytokine responses [11].

The physicochemical characterization of nanoparticles is of paramount importance for tuning and exploiting their properties at the biological level. The morphology, size and size distribution, and surface characteristics (charge, zeta potential, and structure of surface coating) play a key role in nanoparticle distribution in the human body, in protein binding, the interaction with targeting sites, and routes of administration [12]. These characteristics (i.e., size, size distribution, shape, etc.) are deemed to be Critical Quality Attributes and are required to be included in the dossier of the final pharmaceutical product by the regulatory agencies, according to the International Council for Harmonisation (ICH) published guidelines.

Cryogenic Transmission Electron Microscopy (cryo-TEM) is an imaging technique that allows for the identification and study of particles based on their shape, internal structure, and surface characteristics without the use of other methods. Liquid nanodispersions and nanosuspensions can be analyzed, and details about their morphology and shape, size distribution, lamellarity, and inner structural details can be extracted. In some cases, these properties are comparable to the physicochemical characteristics of these liquid nanoformulations analyzed using light and other scattering techniques [9,13,14].

Although the nanoparticle characteristics, such as rigidity, size, and surface charge, can be introduced through well-established means and methodologies, the ability to control the morphology can be a much more challenging prospect. Lipidic and polymeric nanosystems, and thus the hybrid systems from both polymers and lipids, are co-assembled systems; therefore, the co-assembly process and the conditions under which it is taking place strictly affect the resultant morphology [15,16,17]. Typical examples of changes in the nanostructure by controlling the self-assembly process, through molecular engineering, include the formation of spherical or worm-like micelles, vesicles, and other complex morphologies [18,19]. 

Moreover, the careful control of conditions during the preparation process, such as the composition of organic solvents, rate of addition of aqueous solution, temperature cycling, osmotic shock, and chemical cross-linking plays a key role in the morphology differentiation process [20,21]. The vesicular structures observed in mixed nanosystems, composed of both lipids and polymers, exhibit great versatility, meaning the ability to demonstrate, under certain conditions, shape transformation in response to physical factors as a result of the thermodynamic restructuring of polymer chains and surface topology [22,23,24]. 

Other observed morphologies of nanoassemblies, such as discs, compound vesicles, and staggered lamellae, can also be utilized for drug delivery applications and their morphology is found to affect the behavior in the biological environment and processes such as cellular internalization and blood circulation [15,25]. For example, according to the literature, the aspect ratio of rod-like nanoparticles affects cellular internalization, with particle length being a key aspect in differentiating the biological pathways to be followed [26]. Comparative studies, where different morphologies (spherical, discoidal, cylindrical, and hemispherical) of the same biomaterials were injected in vivo, highlighted the differences in organ accumulation characteristics, with discs appearing to avoid the liver to the greatest extent [27]. Excessive particle stiffness is considered to retard cellular uptake, and thus, the addition of plasticizers in the nanoparticulate formulation, like cholesterol, is found to enhance the uptake [28,29]. 

The aim of this investigation is to make a structural evaluation of hybrid lipid–polymer nanoparticles using cryo-TEM. Different kinds of hybrid lipid–polymer nanoparticles were designed and developed using phospholipids and block copolymers with different preparation protocols. The structures obtained ranged from vesicles to rod-shaped structures, worm-like micelles, and irregular morphologies. To the best of the authors’ knowledge, this is the first report in the literature where cryo-TEM is applied for the structure-based characterization of hybrid lipid–polymer nanoparticles and for the connection of their structure with the components of the system, especially the polymeric guest.

## 2. Materials and Methods

### 2.1. Materials

**Figure 1:**
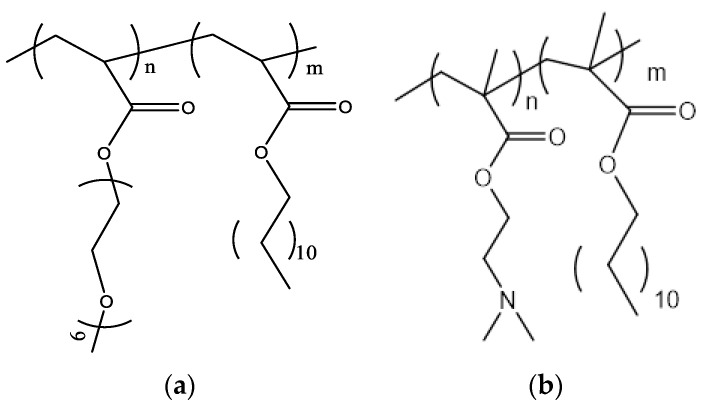
The chemical structures of (**a**) POEGA-PLA and (**b**) PLMA-b-PDMAEMA.

1,2-dipalmitoyl-sn-glycero-3-phosphocholine (DPPC); L-a-phosphatidylcholine hydrogenated (Soy) (HSPC); 1,2-distearoyl-sn-glycero-3-phosphocholine (DSPC); 1,2-dioleoyl-3-dimethylammonium-propane (DAP); dimethyldioctadecylammonium (DDA); and D-(+)-trehalose 6,6’-dibehenate (TDB) were purchased from Avanti Polar Lipids Inc., (Albaster, AL, USA). Chloroform and methanol were purchased from LabScan (Dublin, Ireland). The poly(oligoethylene glycol acrylate)-b-poly(lauryl acrylate) (POEGA-PLA) (Figure 1a) block copolymer was synthesized using RAFT polymerization methodologies. The POEGA block was prepared first and then used as a macromolecular chain transfer agent for the synthesis of the second PLA block. The poly(2-(dimethylamino)ethyl methacrylate)-b-poly(lauryl methacrylate) (PDMAEMA-b-PLMA) (Figure 1b) amphiphilic diblock copolymer was synthesized using RAFT polymerization methodologies [30].

### 2.2. Methods

#### 2.2.1. Preparation of Polymer–Lipid Hybrid Nanoparticles 

Thin-film hydration methods and aqueous heat methods used as previously described [24,25,31,32]. Briefly, for the aqueous heat method, phospholipids/block copolymer were mixed into HPLC-grade water to a concentration of 2 or 5 mg/mL (colloidal concentration), heated to 70 °C (at least 10 degrees °C higher temperature than the temperature above Tm (main transition temperature) of each lipid) for 30 min with a rate of 5 °C/min for 30 min with intermittent resting periods of 5 min (600 rpm using a magnetic stirrer), and then cooled to room temperature at a rate of 5 °C/min [32]. For the thin-film hydration method, desired amounts of phospholipids and block copolymer were dissolved in chloroform/methanol (9:1 *v*/*v*) and then transferred into a round flask connected to a rotary evaporator. A vacuum was applied, and the mixed phospholipids/block copolymer thin film was formed by slow removal of the solvent at 45–50 °C. The mixed film was maintained under vacuum for at least 24 h in a desiccator to remove traces of solvent and, subsequently, was hydrated in HPLC-grade water via slow stirring for 1 h in a water bath above the phase transition temperature of lipids. The resultant nanostructures were subjected to two 5 min sonication cycles (amplitude 70; cycle 0.7) interrupted by a 5 min resting period, in a water bath, using a probe sonicator. The resultant nanostructures were allowed to anneal for 30 min [31].

#### 2.2.2. Cryogenic Transmission Electron Microscopy (Cryo-TEM)

Cryogenic Transmission Electron Microscopy (cryo-TEM) images were obtained using a Tecnai F20 X TWIN microscope (FEI Company, Hillsboro, OR, USA) equipped with a field emission gun, operating at an acceleration voltage of 200 kV. Images were recorded on a Gatan Rio 16 CMOS 4k camera (Gatan Inc., Pleasanton, CA, USA) and processed with Gatan Microscopy Suite (GMS) software version 3.31.2360.0 (Gatan Inc., Pleasanton, CA, USA). Specimen preparation was performed via vitrification of the aqueous solutions on grids with holey carbon film (Quantifoil R 2/2; Quantifoil Micro Tools GmbH, Großlöbichau, Germany). Prior to use, the grids were activated for 15 s in oxygen plasma using a Femto plasma cleaner (Diener Electronic, Ebhausen, Germany). Cryo-samples were prepared by applying a droplet (3 μL) of the suspension to the grid, blotting with filter paper and immediate freezing in liquid ethane using a fully automated blotting device Vitrobot Mark IV (Thermo Fisher Scientific, Waltham, MA, USA). After preparation, the vitrified specimens were kept under liquid nitrogen until they were inserted into a cryo-TEM-holder Gatan 626 (Gatan Inc., Pleasanton, CA, USA) and analyzed in the TEM at −178 °C.

#### 2.2.3. Dynamic and Electrophoretic Light Scattering

The physicochemical behavior of the prepared nanosystems was characterized by measuring the size (hydrodynamic radius R_h_, nm) and the size distribution (polydispersity index, PDI) using dynamic light scattering (DLS), while the ζ-potential (ζ-pot, mV) of the nanoparticles was measured by electrophoretic light scattering (ELS). In total, 100 μL of aliquots was 30-fold diluted in HPLC-grade water. Dynamic light scattering measurements were performed using an AVL/CGS-3 Compact Goniometer System (ALV GmbH, Berlin, Germany), equipped with a cylindrical JDS Uniphase 22 mV He–Ne laser, operating at 632.8 nm, and an Avalanche photodiode detector. The system was interfaced with an ALV/LSE-5003 electronics unit for the stepper motor drive and limit switch control and an ALV-5000/EPP multi-tau digital correlator. Autocorrelation functions were analyzed using the cumulants method and the CONTIN. Apparent hydrodynamic radii, R_h_, was calculated at finite concentrations using the Stokes–Einstein equation:

(1) $$R_{h}=\frac{k_{B}T}{6\pi n_{0}D}$$

where k_B_ is the Boltzmann constant, n_0_ is the viscosity of water at temperature T, and D is the diffusion coefficient at a fixed concentration. The polydispersity of the particle sizes was given as the μ_2_/Γ^2^ (PDI) from the cumulants method, where Γ is the average relaxation rate, and μ_2_ is its second moment. For the ELS, 100 μL of aliquots was 30-fold diluted in HPLC-grade water, and ζ-potential was measured at room temperature at 633 nm. The ζ-potentials were calculated from electrophoretic mobilities using the Henry correction of the Smoluchowski equation. More details regarding the DLS [24] and ELS [24,31] protocols are described in detail in our previous studies. The R_h_, PDI, and ζ-pot values of the nanoparticles were averaged from triplicate measurements, and the results were reported as a mean ± standard deviation.

## 3. Results and Discussion

### 3.1. Hybrid Nanoparticles Employing POEGMA-PLA

The DPPC/HSPC:POEGMA-PLA polymer–lipid hybrid nanoparticles were prepared using the thin-film hydration method followed by probe sonication as a size reduction method. In both cases of hybrid nanoparticles with POEGMA-PLA, the size of these polymer–lipid hybrid nanoparticles was at the nanoscale (below 100 nm) and the size distribution was as narrow as the PDI values indicated (Table 1).

**Figure 2:**
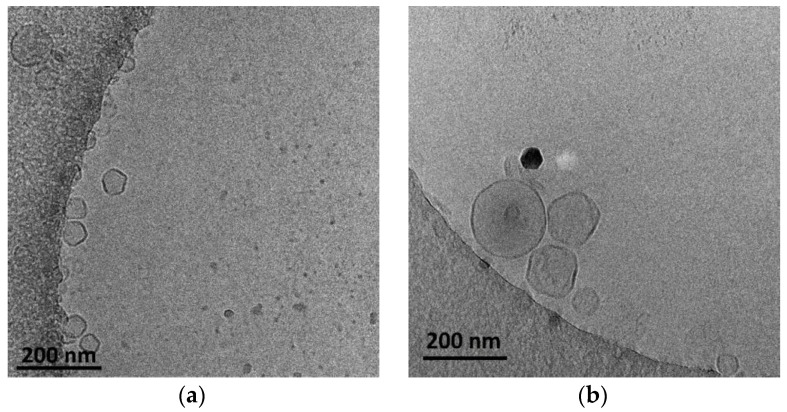
Cryo-TEM images of (**a**) DPPC:POEGMA-PLA (9:0.2 weight ratio) and (**b**) HSPC:POEGMA-PLA (9:0.2 weight ratio) polymer–lipid hybrid nanoparticles. The images for all systems were taken at the same point in time, immediately after preparation for comparison reasons.

The presence of HSPC led to a slightly higher hydrodynamic radius (R_h_) of the nanoparticles, probably due to the higher main transition temperature (Tm) of this phospholipid in comparison to DPPC. The Tm is considered a key parameter for the preparation of lipid-based nanoparticles because it governs the self-assembly process during the hydration of the thin film. The cryo-TEM images showed nanoparticles that were mainly vesicular and unilamellar, with inhomogeneous incorporation of the POEMA-PLA copolymer into the lipid bilayer (Figure 2).

**Figure 3:**
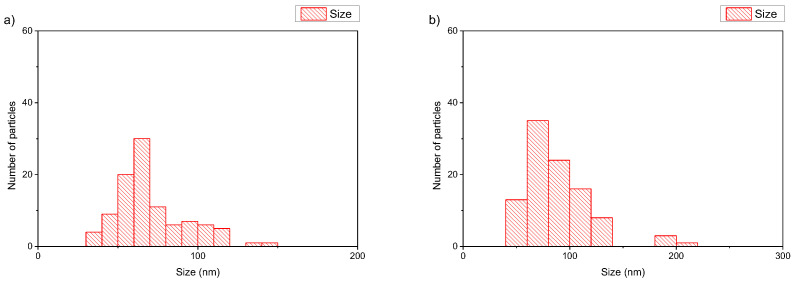
Histograms of (**a**) DPPC:POEGMA-PLA 9:0.2 and (**b**) HSPC:POEGMA-PLA 9:0.2 polymer–lipid hybrid nanoparticles.

The size of the polymer–lipid hybrid nanoparticles is almost the same as the values observed by DLS (Table 1). The thickness of the hybrid membrane was found to be between 6 and 8 nm, which in all cases was higher in comparison to pure liposomal membranes [33]. In particular, in the case of DPPC:POEGMA-PLA 9:0.2 (Figure 2a), cryo-TEM revealed spherical or irregular-shaped particles with sizes in the range of 30–150 nm and a membrane thickness of ~6 nm [33]. The HSPC:POEGMA-PLA polymer–lipid hybrid nanoparticles exhibited a larger size and an inhomogeneous distribution in comparison to the DPPPC:POEGMA-PLA. In other words, replacement DPPC with HSPC leads to the formation of spherical or irregular-shaped particles (Figure 2b) with sizes in the range of 40–215 nm and a membrane thickness of ~6 nm. The latter means that the selection of the lipid is important for altering the morphological and physical properties of polymer–lipid hybrid nanoparticles. The size distribution is presented in Figure 3, and stability study over time is presented in Figure S1, confirming their colloidal stability. Histograms were prepared by measuring 100 particles from cryo-TEM images. The number of particles in the histograms depends on the particle concentration. Generally, the more particles measured, the higher the accuracy of the histograms. We tried to measure at least 100 particles, although this is not always possible due to their low concentration in the sample. Pure polymeric nanostructures of POEGMA-PLA in the form of micelles were prepared in water and exhibited a mean hydrodynamic diameter of 32 nm with PDI at 0.21, thus being smaller in comparison to the mixed nanostructures with lipid.

### 3.2. Hybrid Nanoparticles Employing PLMA-b-PDMAEMA

The aqueous-heat method was used for the preparation of DSPC:DAP:PLMA-b-PDMAEMA nanosystems at 1:0.7:0.03 and 1:1:0.03 weight ratios, without any other method for the size reduction of the systems. The colloidal concentration was equal to 2 mg/mL. In this case, we kept the weight ratio stable for the zwitterionic lipid DSPC/polymer and increased the weight ratio for the cationic lipid DAP. The increase in DAP led to a more homogenous population of particles with a smaller size and higher positive (as expected) values of zeta potential. The DSPC:DAP:PLMA-b-PDMAEMA 1:1:0.03 weight ratio polymer–lipid hybrid nanoparticles were also at the nanoscale. The last observation is quite important because we achieved these physicochemical characteristics without using any size reduction method. The cryo-TEM images showed two kinds of species: unilamellar-vesicular and rod-like structures. 

**Figure 4:**
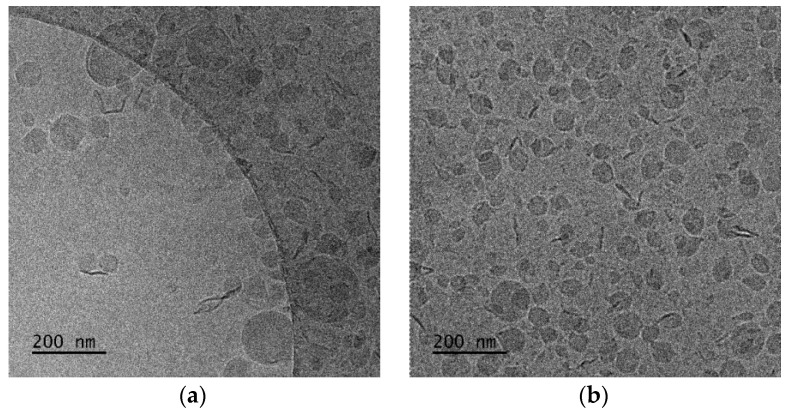
Cryo-TEM images of DSPC:DAP:PLMA-b-PDMAEMA polymer–lipid hybrid nanoparticles: (**a**) 1:0.7:0.03 and (**b**) 1:1:0.03 wt ratios. The images for all systems were taken at the same point in time, immediately after preparation for comparison reasons.

**Figure 5:**
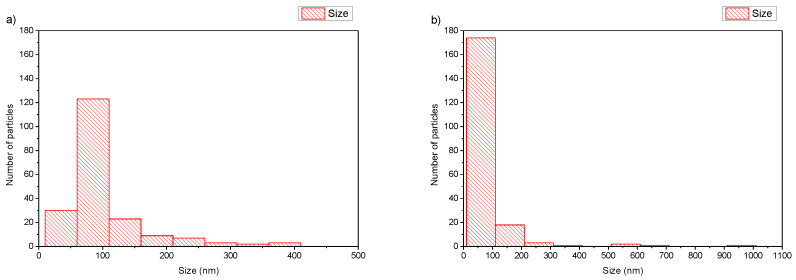
Histograms of DSPC:DAP:PLMA-b-PDMAEMA polymer–lipid hybrid nanoparticles: (**a**) 1:0.7:0.03 and (**b**) 1:1:0.03 wt ratios.

In the case of DSPC:DAP:PLMA-b-PDMAEMA 1:0.7:0.03 wt ratio (Figure 4a), cryo-TEM revealed the predominant spherical or irregular-shaped particles with sizes in the range of 30–410 nm. As shown in Figure 4, there is a significant difference in the particle sizes ranging from 100 nm to 400 nm for DSPC:DAP:PLMA-b-PDMAEMA 1:0.7:0.03 weight ratio and between 100 nm and 1000 nm for DSPC:DAP:PLMA-b-PDMAEMA 1:1:0.03 weight ratio polymer–lipid hybrid nanoparticles. On the other hand, as shown in Figure 5, the main population of the nanoparticles exhibits a size of around 100 nm. This phenomenon is strongly associated with the preparation protocol of hybrid particles. As mentioned before, we did not use any size reduction method (i.e., extrusion through polycarbonate filters) in order to achieve extremely low polydispersity. We assume that the obtained high number of the nanoparticles is multifactorial, being due to both the preparation method and the presence of the helper lipid DAP that, acting like a surfactant, increases the inner surface by crumbling the large nanoparticles into smaller ones of a higher population. The self-assembly of these materials is the reason for the size and morphology heterogeneity. The size distribution is presented in Figure 5, and stability study over time is presented in Figure S2, confirming their colloidal stability. Histograms were prepared by measuring 200 particles from cryo-TEM images. There were also rod-like particles observed as a minority component. For DSPC:DAP:PLMA-b-PDMAEMA 1:1:0.03 wt ratio nanosystems, cryo-TEM revealed the predominant spherical or irregular-shaped particles with sizes in the range of 30–300 nm. There was also a small number of particles larger than 300 nm. The size distribution is presented in Figure 5b. The particles were co-existing with rod-like particles. At the lowest weight ratio of DAP, the presence of rod-like structures was observed, and these structures exhibited lengths of around 100 nm. 

According to the literature, the elongated shaped morphologies, like the present rod-like structures, are correlated with an increase in the circulation time due to their elongated form, which performs well in flow and has been shown to prevent phagocytosis through flow-induced shear forces [34]. According to the literature, the aspect ratio of rod-like nanoparticles affects cellular internalization, with particle length having a key role in differentiating the biological pathways taking place. More specifically, the rod-shaped particles have been found to exhibit greater transport across intestinal cells compared to their spherical counterparts, enhancing their accumulation in the desired cells, and thus being ideal for oral drug delivery applications where intestine penetration is crucial for bioavailability enhancement [35]. Toxicity issues due to morphology are also raised. According to the literature, biodistribution studies have highlighted that particle morphology not only affects behavior in the bloodstream but also the localization in various tissues, with elongated particles showing the least accumulation in the kidneys [27]. Thus, we can expect that morphology, being dependent on the lipid–polymer ratio, can also affect the biological behavior of the nanosystems, the nanoparticle-biological system interactions, and their pharmacokinetic and excretion profile. 

The presence of cationic lipid seems to play a key role in the formation of other structures, except spherical ones. Previously, in lower concentrations of DAP, we also observed “spaghetti” structures, which exhibited different physicochemical characteristics [36]. The same structures have also appeared in complex formations between cationic liposomes and nucleic acids [36]. 

Taking into consideration the already existing literature [37], the introduction of the cationic lipid DAP in the lipidic formulation consisting only of DSPC and PLMA-b-PDMAEMA copolymer led to an increase in the grade of organization from spherical or irregular particles with low contrast, with a size range of 50–600 nm, to the present structures of more homogenous morphology and smaller sizes. In our previous work [37], we concluded that an amount of copolymer PLMA-b-PDMAEMA is not fully incorporated in the DSPC formulations, which can contribute to “threadlike” structures that cover the vesicles. In the present work, we observe that the presence of DAP can assist in the better incorporation of the copolymer, transforming the “threadlike” structures into a coexistence of spherical and rod-like particles.

**Figure 6:**
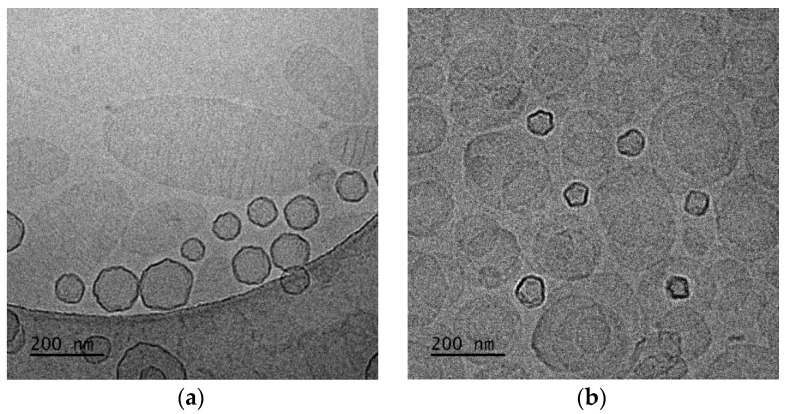
Cryo-TEM images of (**a**) DMPC:PDMAEMA-b-PLMA 95:5 and (**b**) DPPC:PDMAEMA-b-PLMA 95:5 polymer–lipid hybrid nanoparticles. The images for all systems were taken at the same point in time, immediately after preparation for comparison reasons.

We also checked the physicochemical characteristics of polymer–lipid hybrid nanoparticles composed of PDMAEMA-b-PLMA without the presence of cationic lipid. We used zwitterionic lipids with different T_m_, i.e., DMPC with T_m_ at 24 °C and DPPC with Tm at 41 °C. The aqueous heat method was also used as a preparation protocol, and the colloidal concentration was equal to 5 mg/mL. We observed the same trend that we described previously. In particular, the presence of DPPC led to a higher hydrodynamic radius (R_h_) of the nanoparticles and higher PDI values, probably due to the higher main transition temperature (T_m_) of this phospholipid in comparison to DMPC. So, the Tm of the phospholipid seems to be another crucial parameter for the design of hybrid polymer lipid nanoparticles. In contrast, the cryo-TEM images showed the formation of DMPC-based structures with irregular shapes with sizes of around 600 nm, except for the vesicles with a thickness of the hybrid membrane being in the range of 8–10 nm. The DPPC:PDMAEMA-b-PLMA 95:5 weight ratio polymer–lipid hybrid nanoparticles were found to be between 150 and 300 nm in diameter with spherical or polygonal shapes (Figure 6).

**Figure 7:**
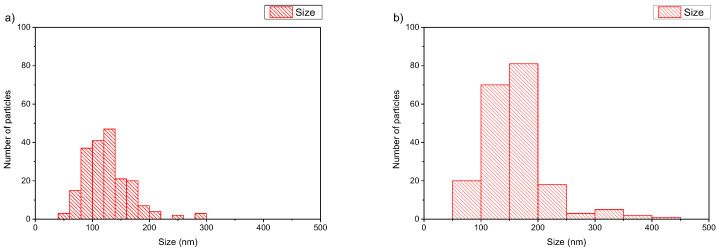
Histograms of (**a**) DMPC:PDMAEMA-b-PLMA 95:5 and (**b**) DPPC:PDMAEMA-b-PLMA 95:5 polymer–lipid hybrid nanoparticles.

In the case of DMPC:PDMAEMA-b-PLMA 95:5 wt ratio (Figure 6a), cryo-TEM revealed the predominant spherical or irregular-shaped particles with sizes in the range of 50–300 nm. The particles have a distinct membrane with a thickness of 7–10 nm. The size distribution is presented in Figure 7a, and stability study over time is presented in Figure S3, confirming their colloidal stability. Histograms were prepared by measuring 200 particles from cryo-TEM images. This large population coexisted with oval or irregular-shaped particles with a striped structure and sizes in the range of 100–600.

From a biological point of view, this morphological diversity of the nanovesicles, being either spherical or irregular-shaped or polygonal-shaped particles, can be a projection of their later biodistribution behavior. According to the literature, the larger morphological diversity is considered to demonstrate a higher specific adhesion and more limited non-specific interactions, while being in the bloodstream, over the more rigid, spherical ones, due to their propensity to adhere to the vascular wall. Moreover, an increase in the circulation time of elongated particles is expected because they can perform well in flow, while phagocytosis is also prevented through flow-induced shear forces [27,34].

Furthermore, it is very interesting, from a physicochemical point of view, to focus a little more on the observed striped elongated morphological pattern of some nanoassemblies. This unique striped pattern of the nanoparticles at the DMPC:PDMAEMA-b-PLMA 95:5 (Figure 6a) ratio resembles images from polymeric poly(ionic liquid) (PIL) nanoparticles [38,39,40], where, independent of nanoparticle size, ellipsoids with interior alternating latitudinal stripes, that is, a “wasp-like” pattern with parallel lamellae, were found. The light and dark lamellae stem from the hydrophobic alkyl chains of both the polymer and the phospholipid chains and the charged polymer backbones, respectively, due to higher electron density compared to that of the carbon atoms. According to Zhang et al. [39] and Klinger et al. [41], with increasing hydrophobicity in longer alkyl chains, their exposure at the surface is energetically unfavorable, and the intraparticle morphologies shift from ellipsoid (wasp-like) to spherical ones, a result of the energy balance of interfacial energy between the hydrophobic alkyl chains and the hydrophilic charged backbones and the surface energy of the particle. This phenomenon can explain the morphological transformation that we observe when the lipid DMPC of 14 carbon alkyl chains is replaced by the lipid DPPC of 16 carbon alkyl chains.

On the other hand, different solid DPPC polymorphs, called ripple and tilt (or gel), are hypothesized to be the source of the patch- and striped-shaped domains, showing that the lower-melting phospholipid can be replaced by an appropriate copolymer to improve mechanical properties while preserving the underlying membrane physics in mixed-phospholipid bilayers when a high-melting phospholipid solidifies on cooling [42]. Apart from this category of particles, there were also rod-like particles observed as a minority component [43]. The replacement of DMPC with DPPC, in prepared hybrids, leads to a different, prevailing morphology of nanosystems (Figure 6b). Predominantly spherical particles were observed with sizes in the range of 50–450 nm and a membrane thickness of 7–8 nm [43]. The size distribution is presented in Figure 7b. This large population coexisted with pentagonal-shaped particles with a diagonal length of 60–100 nm and a wall thickness of ~8 nm. Apart from this category of particles, there were also rod-like particles observed as a minority component.

With a complete picture of the physicochemical and morphological characteristics of polymer–lipid hybrid nanoparticles composed of the amphiphilic block copolymer PLMA-b-PDMAEMA and phospholipids that are widely used for drug delivery purposes, we chose lipids that are widely used for vaccine delivery with adjuvant properties. DDA and TBD are well-established in the literature for their properties in the design and development of subunit vaccines. In this case, we kept the weight ratio of the lipids constant and increased the weight ratio of the polymeric guest with fixed colloidal concentration to 2 mg/mL. The aqueous heat method was used for the formation of the particles without any additional processes for size reduction. The presence of the amphiphilic block copolymer PLMA-b-PDMAEMA at a higher weight ratio led to a decrease in the size of the nanoparticles and a higher homogeneity of the population of the particles. For both systems, the zeta potential values were extremely positive, but we should highlight that the presence of PLMA-b-PDMAEMA seems to cover a small percentage of the positive charge of the lipids, and as the copolymer content increased, the zeta potential values decreased. 

**Figure 8:**
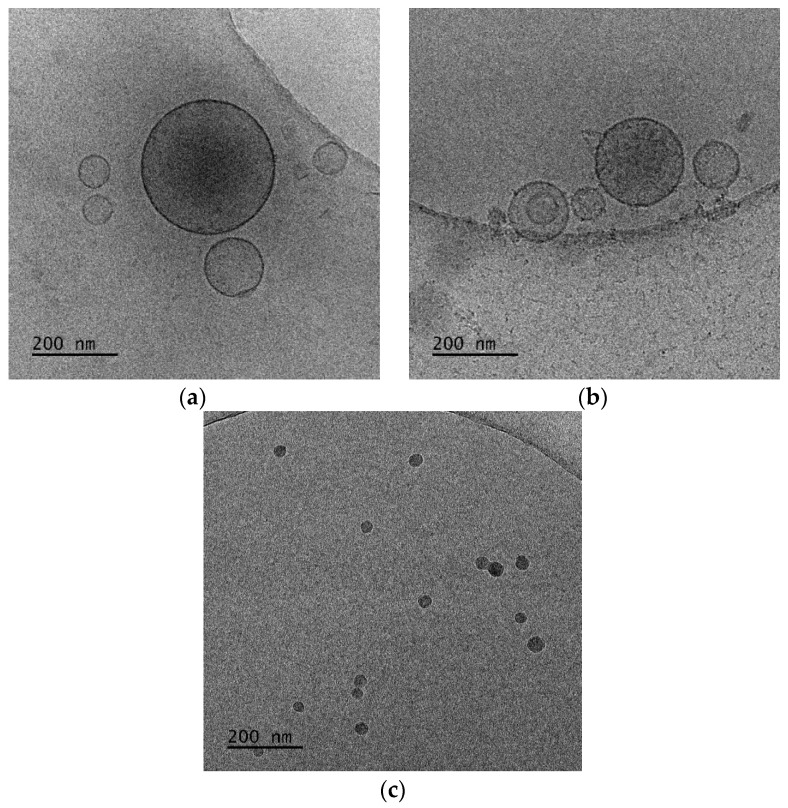
Cryo-TEM images of DDA:TDB:PLMA-b-PDMAEMA polymer–lipid hybrid nanoparticles at (**a**) 1:0.2:1 and (**b**,**c**) 1:0.2:2.5 wt ratios. The images for all systems were taken at the same point in time, immediately after preparation for comparison reasons.

The cryo-TEM images showed spherical and vesicular structures with sizes almost the same as those measured from DLS experiments, taking into account the polydispersity of the systems [44]. In the recent literature, the giant hybrid unilamellar vesicles, as we observed for our systems, have also been called hybridosomes [44]. For DDA:TDB:PLMA-b-PDMAEMA 1:0.2:1 nanosystems (Figure 8a), cryo-TEM revealed spherical vesicles and irregular-shaped vesicles with sizes in the range of 70-590 nm and a membrane thickness of ~6 nm. For DDA:TDB:PLMA-b-PDMAEMA 1:0.2:2.5 vesicles covered with “threadlike” structures (Figure 8b), sizes in the range of 50–460 nm and a membrane thickness of ~6 nm were observed. This population coexisted with spherical “solid” particles (Figure 8c) with sizes in the range of 20–50 nm, which were sometimes joined together [45]. The concentration of particles in both cases was too low to prepare histograms because the overall colloidal concentration was low, while their study over time is presented in Figure S4, confirming their kinetic stability.

Taking into account the physicochemical results of our previous study [30], where pure polymeric nanostructures of PDMAEMA-b-PLMA in the form of micelles were studied, the pure micelles exhibited a mean hydrodynamic diameter of 88 nm with PDI at 0.04, being larger in comparison to mixed nanostructures with lipid, except for the two formulations with the combination of DDA and TDB lipids.

## 4. Conclusions

In conclusion, the physicochemical features of the lipid/polymer components, like the lipid–polymer molar ratio, the polymeric architecture of the block copolymer, the main transition temperature of the phospholipid utilized, as well as the formulation and preparation protocol parameters, are directly connected with the formation of hybrid lipid/polymer vesicles and the diversity of their morphologies. The structures obtained ranged from spherical vesicles to rod-shaped structures, worm-like micelles, and irregular morphologies. The morphological analysis can be a projection of the posterior nanoparticle behavior after being administered or can define aspects like the route of the administration and stability issues. Therefore, such analysis should be included during the preformulation and formulation process of the drug delivery nanosystems. Finally, taking into account the obtained results and all the aspects resulting from their correlation with the already existing literature, we may conclude that the nanoparticle morphology should be considered a Critical Quality Attribute and be required by the regulatory agencies to be included in the dossier of the final pharmaceutical product. Although the general term “morphology” is already mentioned as a Critical Quality Attribute, different aspects of morphology are also mentioned, depending on the different regulatory authorities. However, some distinctive morphological characteristics of the internal morphology should also be taken into account as additional proposed parameters to be considered for the characterization of nanomedicines. For example, the bilayer characteristics, if a bilayer is present, could be one of them [46]. For example, in cases of lipidic nanoparticles carrying nucleic acids, a highly organized internal morphology with cavities is usually observed. Such distinctive morphological details can be obtained only with highly sensitive illustrative techniques such as cryo-TEM, which are proposed in the present study.

## Figures and Tables

**Table 1:** The physicochemical characteristics of the polymer–lipid hybrid nanoparticles.

System	Weight Ratio	Colloidal Concentration	R_h_ (nm)	PDI	z-Pot (mV)	R_g_/R_h_	Type of Morphology
DPPC:POEGMA-PLA	9:0.2	5 mg/mL	42 ± 2	0.2_5_	3.8 ± 0.9	1.22	spherical or irregular-shaped
HSPC:POEGMA-PLA	9:0.2	5 mg/mL	52 ± 4	0.2_2_	0.2 ± 1.8	1.07	spherical or irregular-shaped
DMPC:PDMAEMA-b-PLMA	95:5	5 mg/mL	66 ± 3	0.2_4_	31.8 ± 1.7	-	spherical or irregular-shaped or polygonal-shaped particles; striped elongated morphologies
DPPC:PDMAEMA-b-PLMA	95:5	5mg/mL	82 ± 13	0.3_5_	32.7 ± 6.2	-	spherical particles, pentagonal-shaped particles, rod-like particles
DSPC:DAP:PDMAEMA-b-PLMA	1:0.7:0.03	2mg/mL	76 ± 5	0.3_4_	28.2 ± 7.2	-	unilamellar-vesicular and rod-like structures
DSPC:DAP:PDMAEMA-b-PLMA	1: 1:0.03	2mg/mL	52 ± 6	0.2_1_	41.4 ± 1.8	-	unilamellar-vesicular and rod-like structures
DDA:TDB:PLMA-B-PDMAEMA	1:0.2:1	2mg/mL	151 ± 21	0.4_2_	48.1 ± 2.7	-	spherical vesicles and irregular-shaped vesicles
DDA:TDB:PLMA-B-PDMAEMA	1:0.2:2.5	2mg/mL	102 ± 10	0.3_2_	39.3 ± 1.6	-	“threadlike” structures; spherical “solid” particles

## Data Availability

Data are contained within the article and supplementary materials.

## Supplementary Materials

The following supporting information can be downloaded at: https://www.mdpi.com/article/10.3390/polym16020290/s1, Figure S1: Stability studies over time of (a) R_h_ (nm) and (b) PDI of lipid–POEGMA-PLA systems; Figure S2: Stability studies over time of (a) R_h_ (nm) and (b) PDI of lipid–PDMAEMA-b-PLMA systems; Figure S3: Stability studies over time of (a) R_h_ (nm) and (b) PDI of DSPC:DAP:PDMAEMA-b-PLMA systems; Figure S4: Stability studies over time of (a) R_h_ (nm) and (b) PDI of DDA:TDB:PDMAEMA-b-PLMA systems.
