# Supplementary material for: Structure-Based Evaluation of Hybrid Lipid–Polymer Nanoparticles: The Role of the Polymeric Guest
Source: Polymers (Basel). 2024 Jan 20;16(2):290. doi: 10.3390/polym16020290 (PMC10818590; doi:10.3390/polym16020290)
Supplement: Supplementary file 1 [file polymers-16-00290-s001.zip › polymers-2807099-supplementary.pdf]

SUPPLEMENTARY MATERIAL

Structure-based evaluation of hybrid lipid-polymer nanoparticles: the role of the polymeric guest

Maria Chountoulesi, Natassa Pippa, Aleksander Forys, Barbara Trzebicka and Stergios Pispas

a.

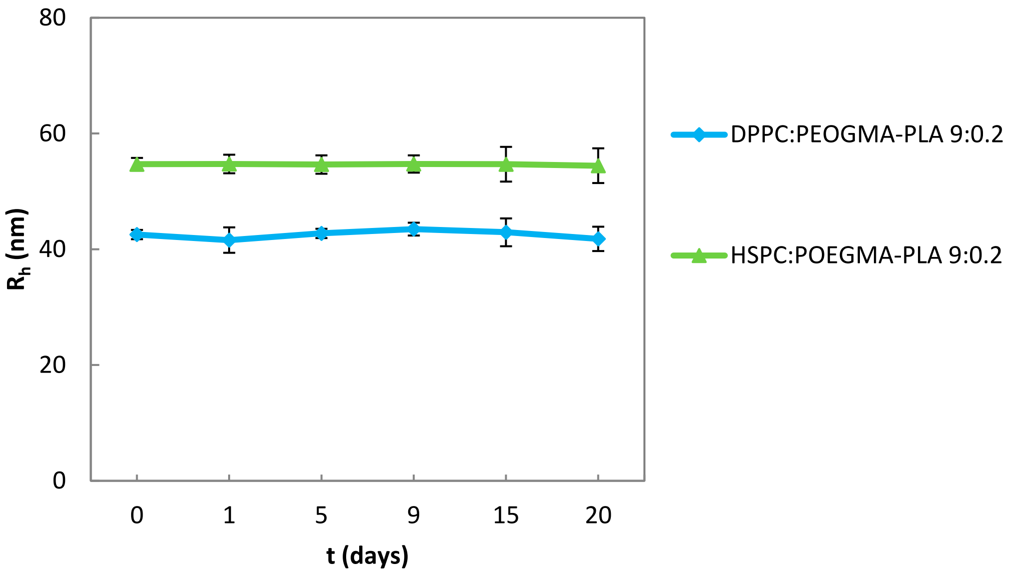

b.

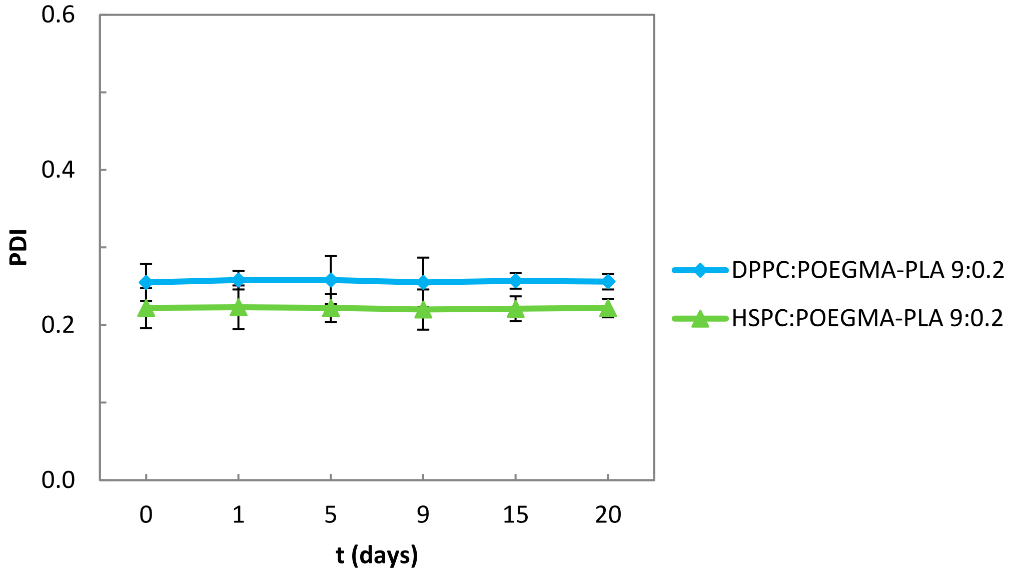

Figure S1: Stability studies over time of **a.** R_h (nm) and **b.** PDI of lipid:POEGMA-PLA systems.

a.

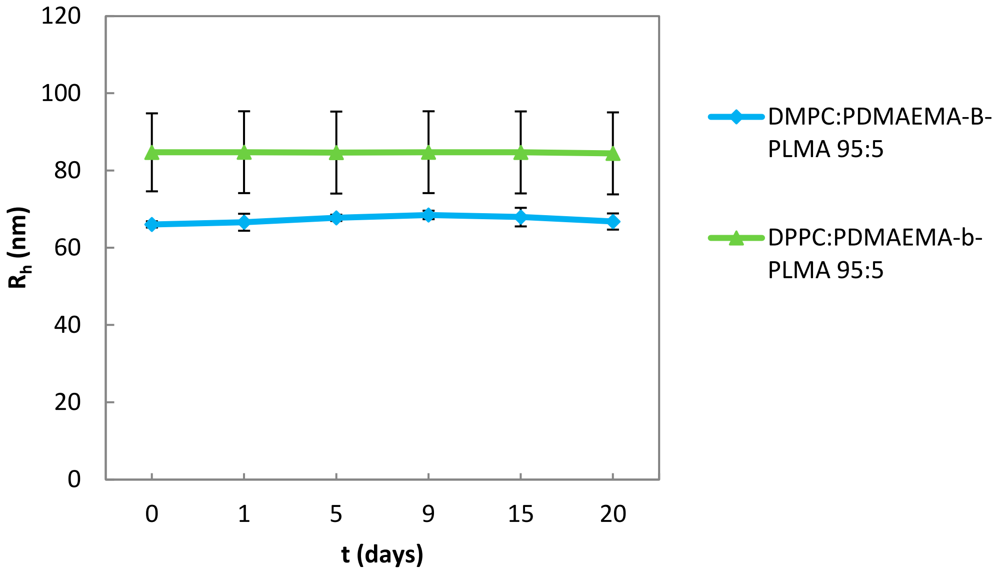

b.

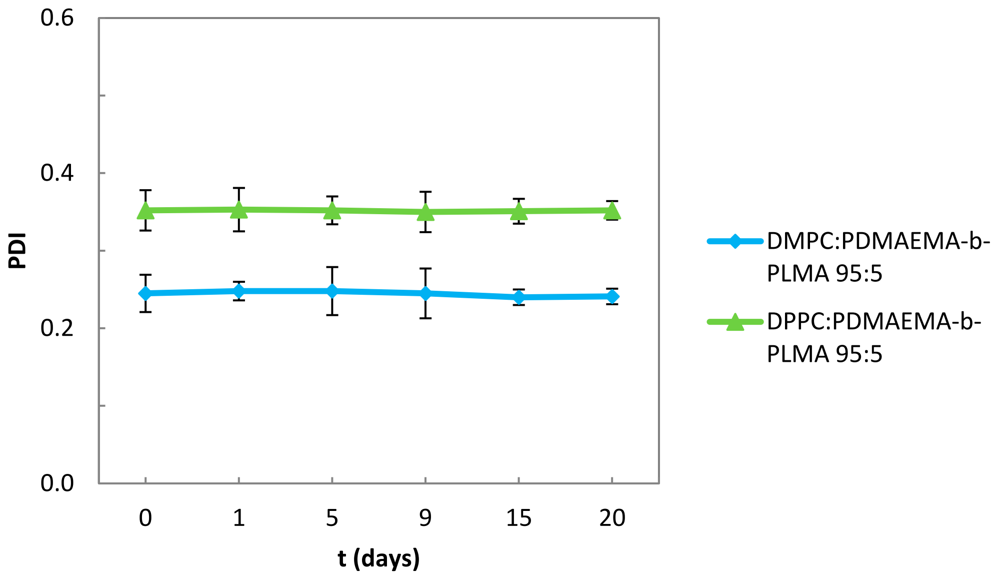

Figure S2: Stability studies over time of **a.** R_h (nm) and **b.** PDI of lipid:PDMAEMA-b-PLMA systems.

a.

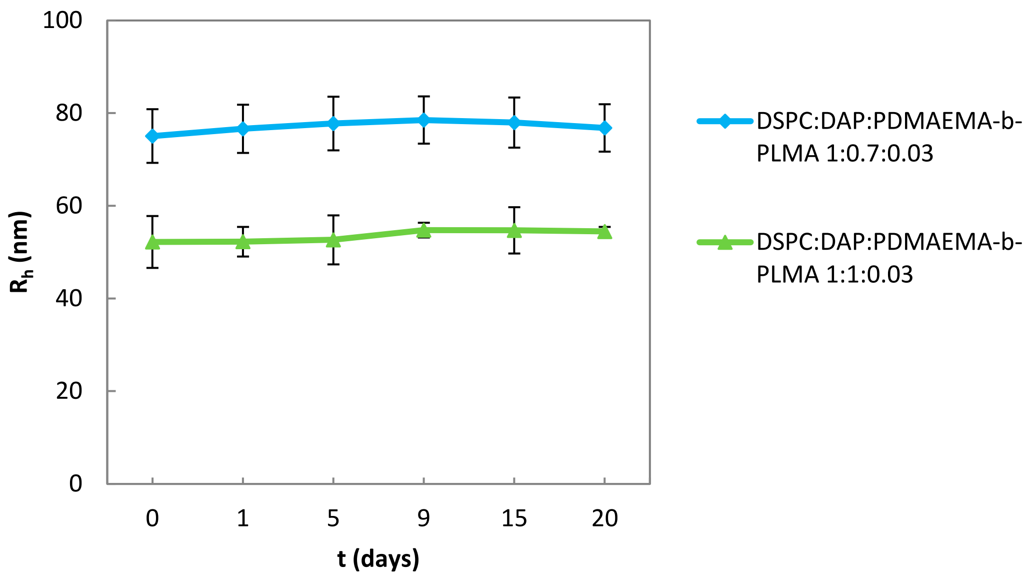

b.

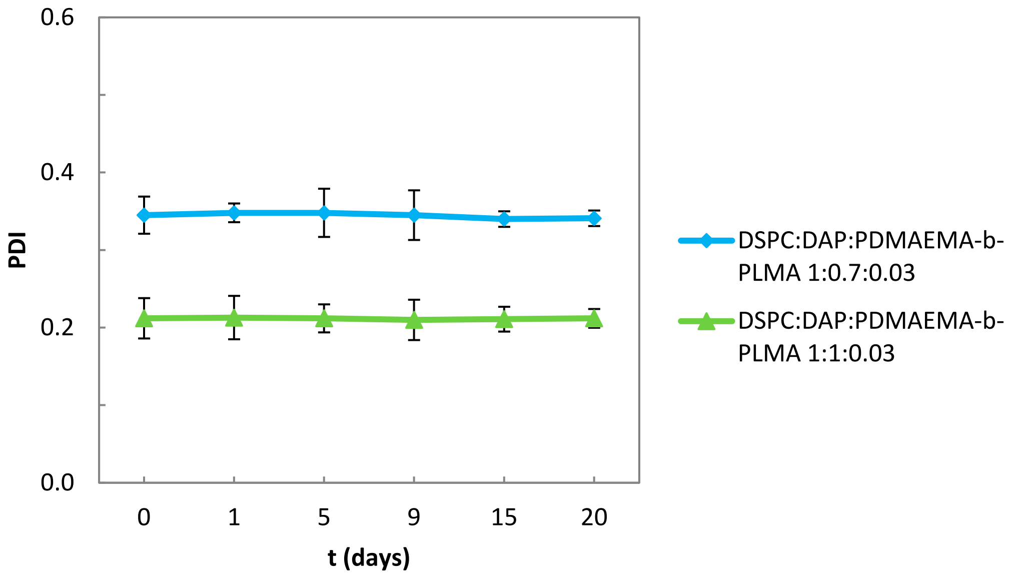

Figure S3: Stability studies over time of **a.** R_h (nm) and **b.** PDI of DSPC:DAP:PDMAEMA-b-PLMA systems.

a.

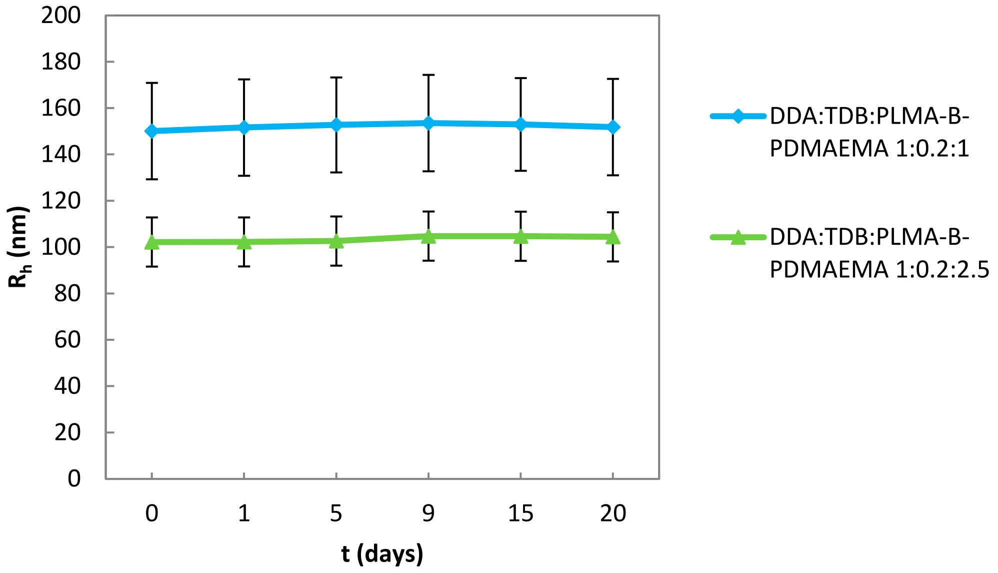

b.

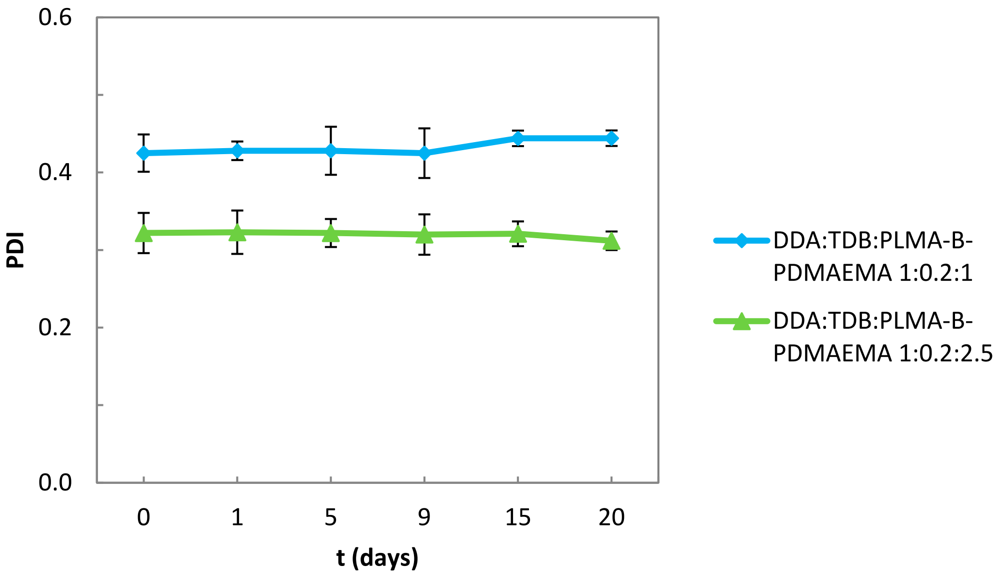

Figure S4: Stability studies over time of **a.** R_h (nm) and **b.** PDI of DDA:TDB:PDMAEMA-b-PLMA systems.
